# Comparison of obesity and metabolic syndrome prevalence using fat mass index, body mass index and percentage body fat

**DOI:** 10.1371/journal.pone.0245436

**Published:** 2021-01-14

**Authors:** Joseph C. Wong, Sheila O’Neill, Belinda R. Beck, Mark R. Forwood, Soo Keat Khoo

**Affiliations:** 1 Department of Nuclear Medicine, Royal Brisbane and Women’s Hospital, Brisbane, Queensland, Australia; 2 School of Clinical Medicine, University of Queensland, Brisbane, Queensland, Australia; 3 Betty Byrne Henderson Women's Health Research Centre, Royal Brisbane and Women’s Hospital, Brisbane, Queensland, Australia; 4 ANU Medical School, Australian National University, Canberra, Australian Capital Territory, Australia; 5 School of Allied Health Sciences & Menzies Health Institute Queensland, Griffith University Gold Coast campus, Gold Coast, Queensland, Australia; 6 The Bone Clinic, Brisbane, Queensland, Australia; 7 School of Medical Science & Menzies Health Institute Queensland, Griffith University Gold Coast campus, Gold Coast, Queensland, Australia; 8 Faculty of Medicine, University of Queensland, Brisbane, Queensland, Australia; University of Mississippi Medical Center, UNITED STATES

## Abstract

**Background:**

Accurate obesity classification is important so that appropriate intervention can be instituted to modify metabolic risk factors. Commonly utilized body mass index (BMI) and percentage body fat (PBF) are influenced by lean mass whereas fat mass index (FMI) measures only body fat. This study compares the prevalence of obesity and metabolic risk factors with FMI, BMI and PBF using DXA (dual-energy x-ray absorptiometry).

**Methods:**

489 women randomly recruited from the electoral roll were stratified into 4 age groups; 40–49, 50–59, 60–69 and 70–79 years from 2000 to 2001. Clinical data and DXA body composition were obtained. Statistical analyses were performed using Medcalc v15 (Ostend, Belgium) with significance level at p = 0.05 (two-tailed).

**Results:**

There was higher prevalence of obesity using PBF compared to BMI and FMI (p<0.001). This difference was greater from age 50–59 (p<0.05) which may be explained by age-related lean mass loss. PBF over-classified obesity in over 35% of normal and 95% of overweight categories compared to FMI and BMI. BMI has a sensitivity of 78.9% and specificity of 98.3% for obesity using FMI as the standard. BMI under-classified obesity in the overweight category by 14.9% compared to FMI. There was no difference in diabetes, dyslipidemia, hypertension and metabolic syndrome prevalence within the BMI-obesity and FMI-obesity categories (p>0.05).

**Conclusion:**

PBF classified more obesity than BMI and FMI because of its low pre-determined threshold. The greater difference with PBF compared to BMI and FMI from the 50–59 decade onwards can be attributed to age-related lean mass loss. BMI had the lowest sensitivity for obesity diagnosis. BMI under-classified obesity in the overweight category compared to FMI due to its inability to differentiate lean mass. However, there was no significant difference in the prevalence of metabolic risk factors between BMI and FMI-obesity categories indicating that fat location may influence metabolic dysregulation.

## Introduction

Obesity is a rapidly growing threat to the health of populations in an increasing number of countries. Worldwide, more than 1.9 billion or 39% of adults were overweight and of these, over 650 million or 13% were obese in 2016. Obesity has nearly tripled worldwide since 1975 [1].

Obesity is related to metabolic disturbances such as insulin resistance and dyslipidaemia, leading to disorders such as diabetes, hypertension and atherosclerotic disease. It is increasingly recognised that it is not obesity per se as defined by body mass index (BMI), but the presence of increased body fat, its distribution within the body, particularly central body obesity, and dysfunction of the body fat within these deposits, known as ‘sick fat’ or adiposopathy that are associated with the development of diseases [2]. In central obesity, the increase in visceral adipose tissue (VAT) is an important contributor to the metabolic complications of obesity [2]. The cluster of risk factors for atherosclerotic disease and type 2 diabetes which includes hypertension, dyslipidaemia (raised triglycerides and lowered high density lipoprotein cholesterol), raised fasting glucose and central obesity is known as the metabolic syndrome [3].

Obesity is defined by the World Health Organization (WHO) using body mass index (BMI) [1]. BMI is calculated as a person’s weight in kilograms divided by the square of height in meters (kg/m^2^). This index applies to adult men and women and correlates with percentage body fat (PBF) [1, 4]. BMI has been incorporated into clinical practice as an easy on-site anthropometric measure of obesity. However, BMI is limited by the inability of the weight numerator to distinguish between the contribution from fat and soft tissue lean mass, the latter of which includes muscle tissue [1]. Individuals with normal weight but excess body fat would not be diagnosed with obesity. Conversely, individuals with high amount of lean mass, that is, large muscle bulk, would be incorrectly classified with obesity. Another limitation of BMI is that the same criteria are used to classify obesity and overweight irrespective of sex or race [5].

Other available measurements to diagnose obesity including percentage body fat (PBF) and fat mass index (FMI). Bioelectrical impedance (BIA) and dual-energy x-ray absorptiometry (DXA) can be used to derive PBF. However, expressing body fat as a percentage of weight has similar limitations as BMI due to the contribution of lean mass to body weight [5].

DXA can also be used to calculate FMI. Whole body scanning using DXA is able to assess body composition based on a three compartment model, determining total body mass (or body weight) as well as the components of total fat mass, soft tissue lean mass and bone mineral content. FMI is the total body fat divided by height squared. The International Society for Clinical Densitometry (ISCD) has proposed using FMI as an alternative method to define body fat categories [6]. The advantage is that FMI provides a sole index of body fat compared to BMI and PBF which uses body weight. In addition, there are sex- and race-specific reference ranges for FMI which avoids these issues with BMI and PBF [6]. FMI classification thresholds are based on the expected prevalence of overweight and obesity defined by BMI at age 25. DXA is regarded as a reference method for body composition analysis which has been validated against criterion multi-compartment models [7]. DXA has high accuracy and precision, making it useful for tracking changes in body fat [8–10]. In contrast, BIA measurements may be subject to errors due to the hydration state and internal body water distribution [7].

While there are prior studies examining the relationship between PBF and BMI [11–15], and between FMI and BMI [16, 17], there are limited studies assessing body fat classification comparing all three metrics simultaneously in adults and these have used BIA [18, 19]. To our knowledge, there are no reports assessing the performance of FMI, BMI and PBF in adults over an extended age range using DXA.

We also compared the relationship between the prevalence of obesity using BMI, FMI and PBF, and the prevalence of obesity related conditions including dyslipidemia, diabetes, hypertension and metabolic syndrome which represent risk factors predisposing to cardiovascular disease. Metabolic syndrome is defined as the presence of any three out of five risk factors which for our women cohort are: waist circumference ≥ 88 cm, triglycerides ≥150 mg/dL or drug treatment for elevated triglycerides, HDL-C <50 mg/dL or drug treatment for reduced HDL-C, blood pressure ≥130 systolic and/or ≥85 mmHg diastolic or anti-hypertensive drug treatment and fasting blood glucose ≥100 mg/dL or drug treatment for elevated glucose [3]. There is some evidence in the literature that fat mass or FMI predicts these risk factors better than BMI and PBF [16, 20–23].

The aim of this study was to compare the prevalence of obesity and metabolic risk factors using FMI, BMI and PBF derived from DXA in women from 40 to 79 years of age.

## Materials and methods

This study is an observational evaluation of a population-based cohort of urban women of peri-menopausal age and beyond in the city of Brisbane in Australia from 2000 to 2001. This study is part of a larger project assessing health in ageing women. Details of the recruitment and retention methods have been previously described [24]. Age stratified random samples of women in the age groups 40–49 years, 50–59 years, 60–69 years and 70–79 years were recruited. Eligibility was restricted to women who were ambulatory or able to be transported and those available and willing to undergo the clinical assessments and to provide informed consent.

A random sample of women between the ages of 40 to 79 was recruited from the electoral roll. A total of 489 white women were stratified into 4 age groups by decades; 40–49, 50–59, 60–69 and 70–79 years. Clinical history was collected including hypertension, diabetes, dyslipidemia, and cardiovascular and diabetic medications. Fasting blood glucose and lipid profile were obtained. Height was measured in the standing position in bare feet using a wall-mounted stadiometer to the nearest 0.5 cm. Waist circumference was measured at the midpoint between the margin of the lowest palpable rib and the iliac crest to the nearest 0.5 cm, taken at the end of the expiratory phase of tidal breathing while in a relaxed posture as recommended by the WHO STEPwise Approach to Surveillance [25].

Body composition was determined with DXA using a GE Lunar DPX-L densitometer (Madison, WI). Total body mass, total fat mass, and soft tissue lean mass in the arms and legs were obtained. BMI was calculated as the total body mass divided by height squared. PBF is the percentage of total fat mass over total body mass. FMI is the total fat mass divided by height squared. WHO classifies normal weight as BMI at 18.5 to 24.9 kg/m^2^, overweight at 25 to 29.9 kg/m^2^, and obesity at equal to or greater than 30 kg/m^2^ [1]. PBF equal to or greater than 35% for females has been accepted to indicate obesity [11, 18, 26]. With FMI, the cut-offs for females for the normal category are 5 to 9 kg/m^2^; for excess fat, comparable to the overweight criteria by BMI, greater than 9 to 13 kg/m^2^; and for obesity greater than 13 kg/m^2^ [5, 6]. Appendicular soft tissue lean mass index (ALMI) is the sum of arm and leg soft tissue lean mass divided by height squared and provides an assessment of skeletal muscle mass [6]. ALMI was analyzed because muscle mass is an important contributor to body weight which affects BMI and PBF values but not FMI. Division by height squared allows normalization for stature [6, 27]. Our laboratory’s coefficients of variation (CV%) of 2.0% for total body fat mass, 1.2% for total body lean mass and 2.0% for total percentage fat are comparable with those of other laboratories [8, 28].

Ethical approval of the study design was obtained from the Ethics Committee of the Royal Brisbane and Women’s Hospital. All participants provided written informed consent prior to commencement of the study.

### Statistical analysis

Data were presented for the body fat categories for the total cohort using BMI, FMI and PBF based on their respective established thresholds. Obesity prevalence for each method was determined for the total cohort and each of the age decades. A comparison of total and subgroup analyses of obesity classification and metabolic syndrome among the three methods were performed using proportions z-tests. Body fat classifications comparing FMI, BMI and PBF were presented. With 100 participants per age stratum, inter-group differences of ≥33% and pooled analysis differences of ≥18% would be detected with 90% power. Univariate analysis was used to identify potential significant factors, including age, weight, diabetes, dyslipidemia and hypertension, influencing ALMI. Detailed demographic characteristics for the cohort were not available to provide further controls for this analysis. Factors that had a p-value <0.1 were included in the stepwise construction of the multivariate model. Statistical analyses were performed using Medcalc v15 (Ostend, Belgium). Significance level was defined at p = 0.05 (two-tailed).

## Results

There were 489 white women in the cohort; 121 in the 40 to 49 age group, 125 in the 50 to 59 age group, 126 in the 60 to 69 age group and 117 in the 70 to 79 age group. Participant characteristics of the total cohort and in the 4 age groups by decades are provided in Table 1.

**Table 1:** Participant body characteristics (mean and 95% confidence interval) and metabolic risk factors (percentage).

Age decades (years)	40–49	50–59	60–69	70–79	Total
**Participants**	121	125	126	117	489
**Body characteristics**					
**Height (m)**	164.0 (162.9–165.1)	163.3 (162.3–164.3)	161.9 (160.8–163.1)	159.1 (158.0–160.2)	162.1 (161.5–162.7)
**Waist circumference (cm)**	82.2 (79.8–84.7)	83.7 (81.6–85.9)	84.5 (82.6–86.3	84.4 (82.4–86.4)	83.7 (82.7–84.8)
**Total body (kg)**	69.9 (67.0–72.8)	71.8 (69.4–74.3)	70.6 (68.5–72.7)	67.2 (65.1–69.2)	69.9 (68.7–71.1)
**BMI (kg/m**^**2**^**)**	26.0 (24.9–27.0)	26.9 (26.1–27.8)	26.9 (26.2–27.7)	26.5 (25.8–27.3)	26.6 (26.2–27.0)
**FMI (kg/m**^**2**^**)**	10.2 (9.4–11.0)	11.1 (10.5–11.8)	11.3 (10.7–11.8)	11.0 (10.4–11.6)	10.9 (10.6–11.2)
**PBF (%)**	37.6 (36.0–39.1)	40.3 (39.0–41.5)	41.1 (40.0–42.2)	40.6 (39.5–41.8)	39.9 (39.3–40.5)
**ALMI (kg/m**^**2**^**)**	6.4 (6.2–6.6)	6.3 (6.2–6.4)	6.3 (6.1–6.4)	6.1 (6.0–6.2)	6.3 (6.2–6.3)
**Metabolic risk factors**					
**Diabetes**	8 (6.6%)	6 (4.8%)	5 (4.0%)	7 (6.0%)	26 (5.3%)
**Dyslipidemia**	40 (33.1%)	46 (36.8%)	56 (44.4%)	68 (58.1%)	210 (42.9%)
**Hypertension**	18 (14.9%)	37 (29.6%)	61 (48.4%)	70 (59.8%)	186 (38.0%)
**Metabolic syndrome**	5 (4.1%)	16 (12.8%)	30 (23.8%)	26 (22.2%)	77 (15.7%)

BMI, body mass index; FMI, fat mass index; PBF, percentage body fat; ALMI, appendicular lean mass index; CI, confidence interval.

Body fat was categorized with FMI, BMI and PBF using their respective cut-offs and their percentages are provided in Table 2. Using FMI, 39.3% have excess fat and 26.2% have obesity. Using BMI, 37.0% were overweight and 21.9% were in the obesity category. Using PBF, 75.9% of the cohort were classified with obesity. No defined threshold for overweight is available for PBF.

**Table 2:** Body fat classification using BMI, FMI and PBF (n = 489).

Body fat category	FMI	BMI	PBF
**Underweight**	18 (3.7%)	9 (1.8%)	NA
**Normal**	151 (30.9%)	192 (39.3%)	NA
**Excess fat/ overweight**	192 (39.3%)	181 (37.0%)	NA
**Obesity**	128 (26.2%)	107 (21.9%)	371 (75.9%)

BMI, body mass index; FMI, fat mass index; PBF, percentage body fat; NA, No defined thresholds for these categories are available.

**Fig 1:**
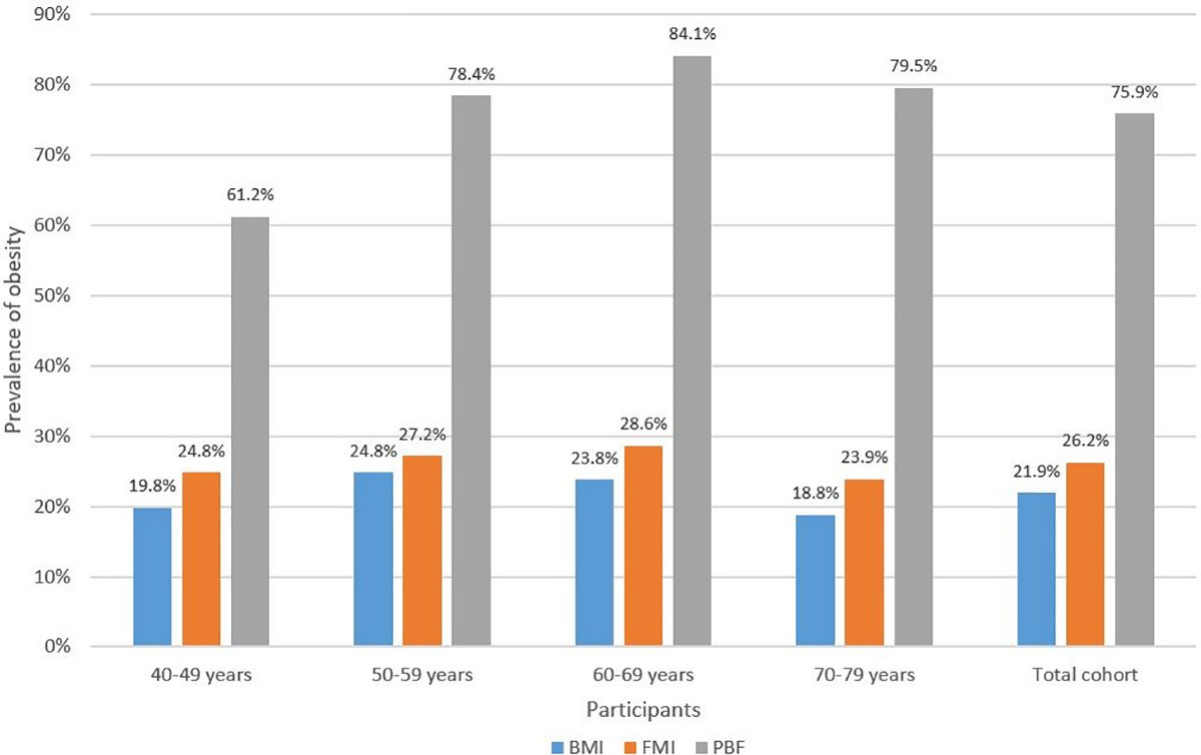
Prevalence of obesity (%) in age decades and total cohort using BMI, FMI and PBF. BMI, body mass index; FMI, fat mass index; PBF, percentage body fat.

Fig 1 shows consistently and markedly higher prevalence of obesity using PBF compared to FMI and BMI in total and subgroup analyses by age decades (p<0.001). The differences between PBF compared to FMI and BMI are significantly greater for the later decades from age 50–59 onwards compared to the 40–49 decade (p<0.05).

Multivariable linear regression model with ALMI as the dependent variable showed increasing age and decreasing weight to be significantly associated with loss of ALMI (p<0.01) (Table 3). Analysis for interaction showed the age:weight interaction term to be significant, with these two factors combined having greater effect on ALMI (p = 0.05). The reduction in ALMI with age would contribute to increases in PBF and decreases in BMI based on their mathematical formulae. The proportions classified using FMI were slightly higher than BMI in total and in all age decades but the differences did not reach statistical significance (p>0.05).

**Table 3:** Multivariable linear regression model with ALMI as the dependent variable for the factors showing significance.

Factor	Estimate	Standard error	Z-score	p-value
**Age**	-0.00902	0.00292	-3.1	<0.01
**Weight**	0.02890	0.00217	13.3	<0.001
**Age:Weight (interaction term)**	-0.00037	0.00190	-2.0	= 0.05

ALMI, appendicular lean mass index.

Based on the FMI categories shown in Table 4, of those categorized with normal FMI, none were classified with obesity using BMI but 35.8% were classified with obesity with PBF. Of those with excess fat on FMI, 3.1% had obesity on BMI and 98.4% on PBF. Of those with obesity based on FMI, 78.9% had obesity on BMI, and 100% on PBF.

**Table 4:** Body fat classification with BMI and PBF compared to FMI.

FMI category	n	Number with obesity using BMI (%)	Number with obesity using PBF (%)
**Normal**	151	0 (0.0%)	54 (35.8%)
**Excess fat**	192	6 (3.1%)	189 (98.4%)
**Obesity**	128	101 (78.9%)	128 (100.0%)

BMI, body mass index; FMI, fat mass index; PBF, percentage body fat.

Based on the BMI categories shown in Table 5, of those categorized with normal BMI, none were classified as having obesity using FMI but 47.9% were classified with obesity on PBF. Of those who were categorized overweight on BMI, 14.9% had obesity using FMI and 95.0% using PBF. Of those with obesity on BMI, 94.4% had obesity using FMI, and 100% with PBF.

**Table 5:** Body fat classification with FMI and PBF compared to BMI.

BMI category	n	Number with obesity using FMI (%)	Number with obesity using PBF (%)
**Normal**	192	0 (0.0%)	92 (47.9%)
**Overweight**	181	27 (14.9%)	172 (95.0%)
**Obesity**	107	101 (94.4%)	107 (100.0%)

BMI, body mass index; FMI, fat mass index; PBF, percentage body fat.

Table 6 shows comparison of the prevalence of diabetes, dyslipidemia, hypertension and metabolic syndrome within the obesity categories using BMI, FMI and PBF. There were no significant differences in the prevalence of these conditions between use of BMI and FMI (p>0.05). The prevalence of metabolic syndrome using PBF was significantly lower than BMI and FMI (p<0.05), with no significant difference for diabetes, dyslipidemia and hypertension (p>0.05).

**Table 6:** Prevalence of metabolic risk factors within obesity classifications using BMI, FMI and PBF (n = 489).

Method	n	Diabetes	Dyslipidemia	Hypertension	Metabolic syndrome
**BMI**	107	10 (9.0%)	48 (44.9%)	57 (53.3%)	33 (30.8%)
**FMI**	128	9 (7.0%)	52 (40.6%)	68 (53.1%)	36 (28.1%)
**PBF**	371	21 (5.7%)	150 (40.4%)	160 (43.1%)	69 (18.6%)

BMI, body mass index; FMI, fat mass index; PBF, percentage body fat

## Discussion

Identification of an accurate method to diagnose obesity is important so that appropriately targeted intervention strategies such as dietary education and control, physical activity programmes, pharmacotherapy and bariatric surgery can be instituted to modify the associated metabolic and atherosclerotic risk factors [29].

This study compared the body fat classification with DXA using fat mass index (FMI), body mass index (BMI) and percentage body fat (PBF). The highest prevalence of obesity occurred with PBF (75.9%), with prevalence using FMI (26.2%) being slightly higher than BMI (21.9%). Therefore, PBF categorized approximately 2.5 times more obesity and FMI about 20% more compared to BMI. A similar but less marked differential prevalence among the three methods was reported by Peltz et al. [18] using BIA with values of 53.5%, 46.7% and 26.3% respectively which may be due to their younger participants.

Our data stratification into age decades showed the differential prevalence pattern was maintained through the age decades from 40–49 to 70–79, with the gap between PBF and both BMI and FMI significantly widening from the 50–59 age decade and persisting thereafter. Multivariate analysis shows significant decline of ALMI with age, which has been described by other studies [30, 31] and would contribute to this gap by increasing PBF and decreasing BMI but not affecting FMI based on their mathematical formulae.

The cut-offs for PBF markedly over-classified obesity in non-obesity categories of both FMI and BMI, with over 35% of normal categories having obesity and over 95% of overweight categories having obesity. This pattern of over-classification of obesity with PBF was similarly observed by Peltz et al. [18]. This may be explained by the selected PBF threshold of 35% for obesity in women being too low. Gallagher et al. [32] and Heo et al. [33] suggested a revised higher obesity PBF cut-off of 40% for white females by linking predicted PBF to WHO guidelines which will lower the percentage of obesity classification.

Assuming FMI as the reference standard based on its sole metric of fat uninfluenced by lean mass, BMI over-classified 3.1% of the sample as having obesity in the FMI-excess fat category, but there was a higher rate of obesity under-classification at 21.1% in the FMI-obesity category. Therefore, there is a greater issue with lower test sensitivity for diagnosis of obesity using BMI at 78.9%, with better specificity at 98.3%. There are no current data in the literature on test sensitivity and specificity results of BMI compared to FMI. However, data exists for comparison of BMI to PBF. A large meta-analysis documented a pooled sensitivity of 50% and specificity of 90% for BMI in detecting obesity when compared to PBF [13]. Similarly, data from NHANES III (National Health and Nutrition Examination Survey III) comparing BMI to PBF also showed low sensitivity in both men (36%) and women (49%) and high specificity in men (95%) and women (99%) for diagnosing obesity [11]. Our data on BMI when compared to PBF showed a low sensitivity at 28.9% and high specificity at 100%. These data support the findings that whereas the BMI cut-off value for obesity has high specificity, it misses a substantial proportion of people with high FMI or PBF.

Assessing categorization with the currently widely used BMI, FMI classified 14.9% as having obesity within the BMI-overweight category, and 5.6% without obesity within the BMI-obesity category. The higher under-classification percentage in the overweight category has been documented and can be attributed to the inability of BMI to differentiate between fat mass and lean mass within this intermediate category [11, 19]. The advantage of FMI is that this metric is not confounded by lean mass.

Comparison of the prevalence of dyslipidemia, diabetes, hypertension and metabolic syndrome within the obesity category showed no significant difference between the use of BMI and FMI (p>0.05), contrary to findings in other studies [16, 20–23]. While FMI overall classified about 20% more subjects with obesity compared with BMI, this did not translate to increased prevalence of dyslipidemia, diabetes, hypertension and metabolic syndrome. There may be several reasons for this. Obesity with metabolic abnormalities are characterized by excess VAT, whereas obesity with a normal metabolic risk profile have low levels of VAT and greater subcutaneous adipose tissue (SAT) [34]. Therefore, it is the distribution of fat within the body, particularly central body obesity [4], and dysfunction of the body fat within these deposits, known as ‘sick fat’ or adiposopathy that are associated with the development of a dysmetabolic state [2]. While FMI may diagnose more obesity, it is not able to differentiate between VAT and SAT locations. It is possible that the FMI-obesity categorized individuals who are BMI-overweight have predominantly excess SAT. Further investigations into the relationship between FMI and metabolic syndrome is required. Our overall prevalence of metabolic syndrome was low at 15.7% compared to approximately 25% with increasing prevalence in advanced age [35]. This may be due to the healthier state of the individuals agreeing to participate in our study. The prevalence of disease affects the positive and negative predictive values for obesity classification for metabolic syndrome, and therefore may not be applicable to the general population.

The observation that the prevalence of metabolic syndrome using PBF-derived obesity being significantly less than BMI or FMI can be explained by the low PBF threshold of 35% for obesity resulting in a much higher prevalence of obesity as discussed previously. As a consequence, PBF-classified obesity is less discriminatory, that is, has a lower positive predictive value, when used to identify co-existing dysmetabolic conditions.

The strengths of this study are the randomized sampling of participants and the use of DXA which has high accuracy and precision in body composition analysis. The limitation is the restriction of the study to mature white women which means it may not extrapolate to younger women, men and other races, although it is anticipated that the same underlying measurement principles relating to relative impact of lean mass on calculations for BMI, FMI and PBF would apply.

## Conclusions

PBF classified more individuals with obesity compared to BMI and FMI, with greater classification disparity from the 50–59 age decade onwards, which may be explained by age-related decline in muscle mass. The high obesity prevalence using PBF and low positive predictive value for metabolic syndrome can be attributed to its low pre-determined percentage threshold for obesity. BMI has a lower sensitivity for obesity diagnosis than FMI or PBF. In particular, the under-classification of obesity with BMI is higher in the overweight category due to its inability to differentiate between fat mass and lean mass compared to FMI. While FMI has the advantage of providing a sole measurement of fat, no difference was found in the prevalence of dyslipidemia, diabetes, hypertension and metabolic syndrome between BMI and FMI categorized obesity states which may indicate that the specific location of fat rather than the amount of fat is important in metabolic dysregulation. Further investigations into FMI and metabolic syndrome are required.

## Supporting information

S1 Appendix Participant data. (XLSX)

S2 Appendix Participant questionnaire. (PDF)
