# Supplementary material for: Comparison of obesity and metabolic syndrome prevalence using fat mass index, body mass index and percentage body fat
Source: PLoS One. 2021 Jan 14;16(1):e0245436. doi: 10.1371/journal.pone.0245436 (PMC7808627; doi:10.1371/journal.pone.0245436)
Supplement: S2 Appendix — (PDF) [file pone.0245436.s002.pdf]

Betty Byrne Henderson
Women's Health Research Centre

Royal Brisbane and Women's Hospital

Body Composition Study

# - Questionnaire -

Office Use

ID No: ……………………………………..

Date returned: ……………………...

Your Name: ……

Your Address: ……

……………………………………………………………………… Postcode: ……………………………

Your Telephone Number:

Home: ………………………………… Work: ………………………………… Mobile: ………………………………….

Email address: ……

Please indicate by ticking the boxes the health condition you have.					
Heart			Diabetes		
0	No	☐	0	No	☐
1	Heart attack (coronary/ infarct)	☐	1	Type 1 (insulin)	☐
2	Ischaemia (angina)	☐	2	Type 2 (late onset)	☐
3	Arrhythmia (rhythm abnormality)	☐			
4	Congestive failure (“weak heart”)	☐	Arterial		
5	Myopathy (heart muscle disease)	☐	0	No	☐
6	Heart valve dysfunction	☐	1	Hypertension on medication	☐
7	Pericarditis (effusion)	☐	2	Peripheral artery disease (legs)	☐
8	Other (state) ……………………………	☐	3	Carotid artery disease	☐
			4	High cholesterol	☐
			5	Other (state) …………………………	☐

MEDICATIONS

Please list any medicines <u>prescribed by your doctor</u> that you are currently using.

1.	2.
3.	4.
5.	6.
7.	8.
9.	10.

Please list any <u>over-the-counter</u> medicines/vitamins you are currently using

1.	2.
3.	4.
5.	6.
